# Relation of hypertension with episodic primary headaches and chronic primary headaches in population of Rafsanjan cohort study

**DOI:** 10.1038/s41598-021-03377-7

**Published:** 2021-12-15

**Authors:** Movahedeh Mohammadi, Fatemeh Ayoobi, Parvin Khalili, Narges Soltani, Carlo La Vecchia, Alireza Vakilian

**Affiliations:** 1 Non-Communicable Diseases Research Center, Rafsanjan University of Medical Sciences, Rafsanjan, Iran; 2 Occupational Safety and Health Research Center, NICICO, World Safety Organization and Rafsanjan University of Medical Sciences, Rafsanjan, Iran; 3 Department of Epidemiology, School of Public Health, Social Determinants of Health Research Centre, Rafsanjan University of Medical Sciences, Rafsanjan, Iran; 4 Department of Clinical Sciences and Community Health, Università degli Studi di Milano (“La Statale”), Via Vanzetti, 5, 20133 Milan, Italy; 5 Department of Neurology, School of Medicine, Rafsanjan University of Medical Sciences, Rafsanjan, Iran

**Keywords:** Ecological epidemiology, Cardiology

## Abstract

Headache has a variety of types, such as episodic primary headaches (EPH) and chronic primary headache (CPH) in its primary form. There is a positive correlation between these two types of headaches and hypertension (HTN), but in some works this correlation has been reported negatively. Therefore, we planned to study HTN-CPH as well as HTN-EPH correlation in our population. A sample of Rafsanjan population (10,000 individuals) entered the cohort study, as one of the Prospective Epidemiological Research Studies in Iran (PERSIAN). We compared the frequency of HTN categories in CPH and EPH cases with a normal population. Out of 9933 participants (46.6% males and 53.4% females) about 29% had EPH and 7.5% had CPH. HTN was found in 24.27% of EPH cases and 31.98% of CPH cases. HTN was also found to be associated with EPH and CPH in the crude model. Two Categories of HTN (Long controlled and uncontrolled) were not associated with EPH. On the other hand, CPH showed associations with all of the HTN categories. After included all variables and confounders, EPH and CPH had association with HTN without any considerable changes. There is strong HTN-EPH as well as HTN-CPH correlations in the studied population.

## Introduction

Headache is one of the most common neurological disorders in any country^1^. Primary headache disorders has been recognized to be consist of migraine and episodic tension type headache, cluster headache, and chronic daily headache consist of chronic tension type headache, medication over use headache, status migraine and other types, which cause some difficulties for patients^2^. According to the studies it could be affected on nearly 3 billion people life’s every year^3–5^. Based on the findings of some studies, headache disorders ranked third out of 369 conditions in terms of years lived with disability (YLDs) for both sexes^5^. Headache disorders tend to be regular and usually become chronic^6^.

About, 10 percent of referrals suffering headache, to clinics of general neurology have been diagnosed with chronic daily headache (CDH), which is usually associated with poor life quality^7^. Many of these patients were underdiagnosed and undertreated^8^. Some studies demonstrated the annual global prevalence of all primary headache disorders about 46%, which 3% was belonged to chronic daily headaches (CDH)^1, 9^. The prevalence of CDH is reported about 2.9% in Asia and almost 4% in Europe^10, 11^. About 10 to 20 percent of people around the world can experience primary episodic headaches during the busiest periods of their working lives^12^. Women suffer from primary episodic headaches four times more often than men^13^.

Chronic primary headache (CPH) is defined as a type of headaches that occurs 15 days a month, for 3 months^6^. This type of headache greatly affect a person’s daily functioning^6, 11^. The conversion from primary episodic headache to chronic headache is often gradual^11^. These patients have significant impairments in their function and quality of life^14^. Therefore, it is important to identify the factors that contribute to the transformation of primary headaches into chronic daily headaches because each of them has different mechanisms in the occurrence of chronic daily headaches^7, 15^. However, the major involved mechanisms on conversion from primary headaches into CDH are still unknown^15^. Overuse of analgesic is recognized as the most important factor for such changes^16^. In addition, Hypertension, allergies, diabetes, obesity and hypothyroidism have been reported to be associated with CDH^17^.

Association between primary headache and various comorbidities has been shown in some studies^18, 19^. Some comorbidity is including: neurological, metabolic and cardiovascular diseases, stroke, epilepsy, multiple sclerosis, obesity, diabetes, and hypertension and sleep disorders^18–20^.

The association between high blood pressure and a headache was first considered in the early twentieth century^21^. It was stated that a throbbing headache in the early morning was a sign of high blood pressure^22^. In this way, the patients who reported the headache as a chief compliant is illustrated more likely to have moderate or severe hypertension than other major complaints^22^. According to the Third Edition of International Classification of Headache Disorders (ICHD), headache related to arterial hypertension were considered only in patients with systolic blood pressure (SBP) ≥ 180 mm Hg and/or diastolic blood pressure (DBP) 120 mm Hg^23^. For many years, many studies attempted to get an association with hypertension or increased BP in migraine^21^. In that way, some large-scale population-based studies reported a positive association between hypertension and migraine^24–26^.

A major risk factor for cardiovascular disease is arterial hypertension (AH), a common disease worldwide^27^. The most generic daily type of primary headache is Tension-type headache (TTH)^2^. Many studies support the hypothesis that TTHs are more susceptible to AH, while people with high BP seem to be at risk for TTH^28^. The relationship between AH and TTH is potentially pathophysiological and clinically significant, but not yet well understood^28, 29^.

The associations between increased BP and headache have been reported repeatedly in the medical literature^30^. Therefore, according to the 3rd edition of International Classification of Headache Disorders (ICHD) and hypertensive patients recording, headache could be considered as the most common symptom in relation to arterial hypertension^23^.

Based on population-based epidemiological studies, there is a relatively stable figure for primary headache disorders in various parts of the world^31^. So that, the last year spread of migraine, episodic tension-type headache, chronic daily headache, chronic tension type headache (CTTH) and medication overuse headache (MOH) are reported approximately 10–12%, 35–68%, 4–5%, 2–3%, 1.5–3% respectively^32^. IHS criteria (1994) and the CDH definition by Silberstein et al. have presented the old definition of headache^33^.

While there is new definition of headaches according to the current version of ICDH3^23^. Chronic daily headache is replaced by chronic primary headache (CPH) in this new classification. Migraine, episodic TTH and episodic TACs are defined as episodic primary headache and CPH consist of chronic migraine, chronic TTH, and chronic TACs^23^. All of these types of headache take long more than 15 days per month and more than 3 months. In TACs type, it takes long more than 1 year attacks and less than 3 months remission^23^. In a study by Caponnet et al. there was a relation between HTN and primary headache^34^.

Previous studies have revealed distinct clinical characteristics for chronic and episodic headaches, including different patterns of response to treatment^35–38^. This difference may indicate varied underlying biological mechanisms, and distinct relationship of chronic vs. episodic headaches with the risk factors. The questionnaires in Rafsanjan cohort study are designed to provide information on the chronicity or episodic nature of the primary headaches; Therefore, this was an opportunity to compare the relationship between primary chronic headaches and its episodic types with hypertension, and to assess the association of headache and its chronicity with blood pressure. To the best of our knowledge this is the first study of this kind.

Considering the importance of following effect on headache, the current study aimed at investigating the association between primary headaches especially episodic versus chronic primary headaches with hypertension based on cohort study of Rafsanjan.

## Methods

### Study design and patient selection

The Rafsanjan cohort study (RCS)^39^ is a part of the prospective epidemiological research studies in Iran (PERSIAN)^40^. The population consisted of 10,000 residents (aged from 35 to 70) of Rafsanjan, a region in the southeast of Iran. 9933 residents were selected out of this population as the eligible to participate in the study. They were interviewed by validated questionnaires^39^. The study protocol was also designed according to the Persian cohort study and approved by the Ethics Committee of Rafsanjan University of Medical Sciences (Ethical codes: ID: IR.RUMS.REC.1399.134).

### Eligibility criteria

We included participants who completed questionnaires information on demography, medical history, habits and laboratory tests.

### Data collection

All participants completed validated electronic questionnaires by interview containing information on demography, medical history, smoking, opium use, medical drug use, alcohol consumption, Body mass index (BMI) (kg/m^2^), family medical history (Diabetes, HTN, cardiovascular disease, stroke, neurological disease, episodic headache, and chronic headache. Moreover, tests were used to measure cholesterol levels, low-density lipoprotein (LDL), high-density lipoprotein (HDL) and triglycerides. Accuracy and precision of all methods were performed in accordance with the relevant guidelines and regulations^39^.

Physical activity (PA) derived from standard PA questionnaire totally calculated as metabolic equivalent of task (MET) for 24 h dependent of metabolic equivalent of activities and were also assessed^40^.

High blood pressure was defined as 140 mm Hg systolic pressure or 90 mm Hg diastolic pressure or higher in each of these levels. The previous diagnosis of hypertension, with or without treatment by antihypertensive drugs was also considered a hypertension case. As our research was a retrospective study, we had all the data about the participants before entering the study. They had been treated using regular or irregular antihypertensive drugs. The patients’ blood pressure was first measured by Richter brand monitor and suitable size of blood pressure cuff and recorded twice-once in each arm-with a 10-min interval. The mean blood pressure of the right arm was used in the analysis^39^.

### Chronicity and control of blood pressure

We also examined duration and control of HTN in participants. Duration of HTN had been already defined as the time interval between self-reported diagnosis of HTN and the date of enrollment. Further, the duration was categorized into ≥ 6 years and < 6 years because 6 years is the median HTN duration in the participants. Controlled HTN also means blood pressure < 140/90 mm Hg.

Accordingly, we classified the subjects into the four below groups:

1. Short duration (< 6 years) controlled HTN (SCH).
2. Short duration (< 6 years) uncontrolled HTN (SUH).
3. Long duration (≥ 6 years) controlled HTN (LCH).
4. Long duration (≥ 6 years) uncontrolled HTN (LUH).

In cohort questionnaire, the status of EPH and CPH was investigated through interview with questions about chronicity and periodicity of headaches after excluding secondary causes such as headaches attributed to head or neck injury, cranial or cervical vascular disorders, non-vascular intracranial disorders, substance use or withdrawal of it, infection, psychiatry disorders (somatization or psychotic disorders), etc. classified as recurrent or episodic primary headaches and chronic primary headaches, and sub classification of primary headaches types was not considered in this interview^41^.

## Statistics

In this study, results were presented using mean, standard deviation (SD), and frequency for both quantitative and qualitative variables. The chi-square test as well as t-test was used to compare categorical and continuous variables between EPH and non-EPH groups. Logistic regression models were also used to investigate the relationships between HTN and the prevalence of EPH and CPH. Based on scientific evidence on primary headache predisposition and the relevant epidemiological literature^12^, confounders were sequentially entered the models according to their hypothesized strengths of association with HTN and EPH and CPH^12^. To reach this goal, the models were first run separately to obtain variables associated with EPH and CPH. The variables whose p-value < 0.2 were considered in multivariate analysis. The first adjusted model included basic socio-demographic characteristics (age, sex, education years, and Occupational status), which were considered to be the most strongly related variables. The second model was adjusted for variables confounding lifestyle (cigarette smoking, alcohol drinking, and opium consumption), physical activity level, and the socio-demographic characteristics to additionally confound HTN-EPH and CPH associations. The third adjusted model included all variables in the second model plus other variables, such as chronic headache family, hypercholesterolemia, body mass index, diabetes mellitus, triglycerides, LDL, and HDL cholesterol. As these variables were potential confounders on the casual pathways linking HTN-EPH and CPH, these three models were created. In all the models, variables of age, education, hypercholesterolemia, body mass index (BMI), triglycerides, LDL, and HDL cholesterol were continuously entered. In addition, the data were analyzed based on duration and control of hypertension in the hypertensive patients.

### Ethics approval and consent to participate

The ethics committee of Rafsanjan University of Medical Sciences approved this study (Ethical codes: ID: IR.RUMS.REC.1399.134). Written informed consent was obtained from the participants. The data of participants kept confidential and was only accessible to the study investigators.

## Results

In this study, 10,000 participants were included in the baseline phase of the RCS. From this population, 9993 participants were eligible for our study. 4622 (46.5%) were males and 5311 (53.5%) were females. Overall, the biological samples of 9941 participants were collected on which laboratory measurements were carried out. The rates of EPH and Chronic primary headaches in the RCS participants were 29.07 (19.20% of men and 37.70% of women) and 7.46% (4.18% of men and 10.32% of women), respectively.

Table 1 shows the socio-demographic characteristics, lifestyle, personal habits, anthropometric measures, clinical risk factors, and blood laboratory assessment of the CPH and EPH groups. There are differences between some socio-demographic characteristics, life style, habits, as well as measured clinical and laboratory indices and other diseases of EPH vs. non-EPH and CPH vs. non-CPH groups (Tables 1, 2).

**Table 1:** Demographic characteristics of participants in the Rafsanjan cohort study.

Characteristics	All (n = 9933)	Chronic primary headache (n = 741)	Non- chronic primary headache (n = 9191)	P-value	Episodic primary headache (n = 2888)	Non-episodic primary headache (n = 7045)	P-value
**Age—year**				0.572			< 0.001
Mean ± SD	49.94 ± 9.56	50.13 ± 9.51	49.92 ± 9.57		48.74 ± 9.21	50.43 ± 9.66	
**Gender—no (%)**				< 0.001			< 0.001
Male	4622 (46.5)	193 (4.18)	4429 (95.82)		886 (19.2)	3736 (80.8)	
Female	5311 (53.5)	548 (10.32)	4762 (89.68)		2002 (37.7)	3309 (62.3)	
**Education—year**				< 0.001			0.004
Mean ± SD	8.51 ± 5.05	7.40 ± 4.93	8.60 ± 5.05		8.61 ± 5.06	8.28 ± 5.00	
**Occupational statues—no (%)**				< 0.001			< 0.001
Unemployed	4143 (41.7)	431 (10.40)	3712 (89.60)		1563 (37.73)	2580 (62.27)	
Retired	1079 (10.9)	63 (5.84)	1016 (94.16)		204 (18.90)	875 (81.10)	
Farmer	737 (7.4)	16 ( (2.17)	721 (97.83)		123 (16.69)	614 (83.31)	
Self-employ	2675 (27)	164 (6.12)	2515 (93.88)		664 (24.82)	2011 (75.18)	
Employment	1295 (13)	67 (5.18)	1227 (94.82)		330 (25.48)	965 (74.52)	
**Cigarette smoking—no (%)**				< 0.001			< 0.001
Yes	2541 (25.7)	123 (4.84)	2418 (95.16)		546 (21.5)	1995 (78.5)	
No	7361 (74.3)	612 (8.31)	6749 (91.69)		2332 (31.7)	5029 (68.3)	
**Alcohol consumption—no (%)**				< 0.001			< 0.001
Yes	1351 (13.6)	64 (4.74)	1287 (95.26)		288 (21.31)	1063 (78.69)	
No	8560 (86.4)	672 (7.85)	7888 (92.15)		2593 (30.30)	5967 (69.70)	
**Opium consumption—no (%)**				< 0.001			< 0.001
Yes	2770 (27.9)	155 (5.60)	2615 (94.40)		616 (22.23)	2154 (77.77)	
No	7142 (72.1)	581 (8.13)	6561 (91.87)		2265 (31.71)	4877 (68.29)	
**BMI**				< 0.001			0.03
Mean ± SD	27.82 ± 4.89	28.67 ± 4.96	27.76 ± 4.88		28.05 ± 4.88	27.73 ± 4.89	
**LDL cholesterol**				0.004			0.52
Mean ± SD	108.06 ± 30.43	111.12 ± 32.68	107.80 ± 30.21		108.99 ± 30.53	107.67 ± 30.35	
**HDL cholesterol**				0.281			< 0.001
Mean ± SD	57.90 ± 12.45	58.38 ± 11.81	57.86 ± 12.51		58.67 ± 12.04	57.59 ± 12.60	
**Triglycerides**				0.066			0.019
Mean ± SD	168.90 ± 109.20	176.07 ± 134.16	168.37 ± 107.09		164.92 ± 103.39	170.59 ± 111.65	
**Cholesterol**				0.003			0.135
Mean ± SD	198.83 ± 41.76	203.30 ± 39.96	198.47 ± 41.89		199.82 ± 38.13	198.44 ± 43.15	
**Physical activity**				< 0.001			0.007
Mean ± SD	38.76 ± 6.35	37.93 ± 4.84	38.86 ± 6.42		38.55 ± 5.32	38.89 ± 6.68	

*BMI* body mass index, *LDL* low-density lipoprotein, *HDL* high-density lipoprotein.

**Table 2:** Selected diseases in relation to chronic primary headache/episodic primary headache among participants in the Rafsanjan cohort study.

Diseases	All (n = 9933)	Chronic primary headache (n = 741)	Non-chronic primary headache (n = 9191)	P-value	Episodic primary headache (n = 2888)	Non-episodic primary headache (n = 7045)	P-value
**HTN—no (%)**				< 0.001			0.007
Yes	2235 (22.5)	237 (10.60)	1998 (89.40)		701 (31.36)	1534 (68.64)	
No	7699 (77.50)	504 (6.55)	7193 (93.45)		2187 (28.41)	5510 (71.59)	
**HTN duration and control—no (%)**				< 0.001			0.079
No	7697 (77.5)	504 (6.55)	7193 (93.45)		2187 (28.41)	5510 (71.59)	
SCH	705 (31.54)	80 (11.35)	625 (88.65)		229 (32.48)	476 (67.52)	
LCH	358 (16.02)	42 (11.73)	316 (88.27)		109 (30.45)	249 (69.55)	
SUH	755 (33.74)	71 (9.42)	683 (90.58)		238 (31.56)	516 (68.44)	
LUH	418 (18.70)	44 (10.53)	374 (89.47)		125 (29.90)	293 (70.10)	
**Diabetes—no (%)**				< 0.001			0.025
Yes	1933 (19.5)	165 (8.54)	1768 (91.46)		522 (27)	1411 (73)	
No	7999 (80.5)	576 (7.20)	7423 (92.80)		2366 (29.57)	5633 (70.43)	
**Cardiac ischemic—no (%)**				0.004			0.073
Yes	870 (8.8)	86 (9.89)	784 (90.11)		230 (26.44)	640 (73.56)	
No	9062 (91.2)	655 (7.23)	8407 (92.77)		2658 (29.33)	6404 (70.67)	
**Myocardial infarction—no (%)**				0.667			0.003
Yes	296 (3)	24 (8.11)	272 (91.89)		63 (21.28)	233 (78.72)	
No	9636 (97)	717 (7.44)	8919 (92.56)		2825 (29.32)	6811 (70.68)	
**Stroke—no (%)**				< 0.001			0.178
Yes	153 (1.5)	27 (17.65)	126 (82.35)		52 (33.99)	101 (66.01)	
No	9779 (98.5)	714 (7.30)	9065 (92.70)		2836 (29)	6943 (71)	
**Chronic headache family—no (%)**				0.396			< 0.001
Yes	829 (8.3)	68 (8.20)	761 (91.80)		319 (38.48)	510 (61.52)	
No	9104 (91.7)	673 (7.39)	8430 (92.61)		2569 (28.22)	6535 (71.78)	

*HTN* hypertension, *SCH* short duration (< 6 years) controlled hypertension, *SUH* short duration (< 6 years) uncontrolled hypertension, *LCH* long duration (< 6 years) controlled hypertension, *LUH* long duration (< 6 years) uncontrolled hypertension.

Moreover, HTN was found in 31.36% of EPH and 10.60% of CPH patients. Also, about half of the hypertensive patients in EPH and CPH groups had controlled and uncontrolled HTN (Table 2).

Further analysis also showed (Table 3) that in the group of patients with hypertension, the type of anti-hypertensive medications had no significant association with headaches.

**Table 3:** Classes of antihypertensive medications in relation to chronic primary headache/episodic primary headache among participants with hypertension in the Rafsanjan cohort study.

Drugs classes	All (n = 2235)	Chronic primary headache (n = 237)	Non-chronic primary headache (n = 1998)	P-value	Episodic primary headache (n = 701)	Non-episodic primary headache (n = 1534)	P-value
**Beta blocker—n (%)**				0.176			0.898
Yes	862 (38.57)	101 (42.62)	761 (38.09)		269 (38.37)	593 (38.66)	
No	1373 (61.43)	136 (57.38)	1237 (61.91)		432 (61.63)	941 (61.34)	
**Calcium channel blocker—n (%)**				0.845			0.859
Yes	470 (21.03)	51 (21.52)	419 (20.97)		149 (21.26)	321 (20.93)	
No	1765 (78.97)	186 (78.48)	1579 (79.03)		552 (78.74)	1213 (79.07)	
**Angiotensin 2 receptor blocker—n (%)**				0.503			0.488
Yes	1121 (50.16)	114 (48.10)	1007 (50.40)		344 (49.07)	777 (50.65)	
No	1114 (49.84)	123 (51.90)	991 (49.60)		357 (50.93)	757 (49.35)	
**Angiotensin-converting enzyme inhibitor—n (%)**				0.527			0.05
Yes	113 (5.06)	14 (5.91)	99 (4.95)		26 (3.71)	87 (5.67)	
No	2122 (94.94)	223 (94.09)	1899 (95.05)		675 (96.29)	1447 (94.33)	
**Thiazide diuretics—n (%)**				0.4			0.798
Yes	399 (17.85)	47 (19.83)	352 (17.62)		123 (17.55)	276 (17.99)	
No	1836 (82.15)	190 (80.17)	1646 (82.38)		578 (82.45)	1258 (82.01)	
**Loop diuretic—n (%)**				0.605			0.122
Yes	55 (2.46)	7 (2.95)	48 (2.40)		12 (1.71)	43 (2.80)	
No	2180 (97.54)	230 (97.05)	1950 (97.60)		689 (98.29)	1491 (97.20)	

Table 4 shows the HTN-EPH as well as HTN-CPH associations using crude and three adjusted models. Various HTN groups are shown in terms of their associations with duration and control of EPH and CPH. It demonstrates that HTN is associated with EPH and CPH in crude model as well as all the adjusted models. Short controlled HTN was also found to be associated with EPH in crude model and in the second as well as the third adjusted. In contrast, Short uncontrolled HTN was not connected with EPH in crude model; however, it was found to be linked with EPH in all the three adjusted models. On the other hand, long controlled and uncontrolled models were not associated with EPH. Considering CPH, it was associated with all HTN categories in crude and all the adjusted models.

**Table 4:** Associations between hypertension with episodic primary headache and chronic primary headache.

	Crude model	Adjusted model 1	Adjusted model 2	Adjusted model 3
	OR (95% CI)^a^	OR (95% CI)^b^	OR (95% CI)^c^	OR (95% CI)^d^
**Episodic primary headache**				
HTN (present vs absent)	1.15 (1.04–1.28)	1.18 (1.04–1.31)	1.22 (1.10–1.40)	1.26 (1.12–1.43)
HTN duration and control				
SCH vs no HTN	1.21 (1.03–1.43)	1.18 (0.98–1.40)	1.21 (1.02–1.45)	1.25 (1.04–1.49)
SUH vs no HTN	1.16 (0.10–1.40)	1.23 (1.04–1.50)	1.32 (1.10–1.60)	1.35 (1.13–1.61)
LCH vs no HTN	1.10 (0.98–1.36)	1.10 (0.85–1.40)	1.13 (0.90–1.45)	1.16 (0.90–1.48)
LUH vs no HTN	1.07 (0.87–1.33)	1.10 (0.90–1.40)	1.16 (0.92–1.47)	1.20 (0.95–1.52)
**Chronic primary headache**				
HTN (present vs absent)	1.69 (1.44–1.99)	1.51 (1.26–1.82)	1.50 (1.24–1.81)	1.52 (1.25–1.85)
HTN duration and control				
SCH vs no HTN	1.83 (1.42–2.34)	1.62 (1.24–2.10)	1.61 (1.23–2.09)	1.66 (1.27–2.17)
SUH vs no HTN	1.48 (1.14–1.93)	1.38 (1.05–1.82)	1.37 (1.03–1.81)	1.36 (1.03–1.80)
LCH vs no HTN	1.90 (1.36–2.65)	1.65 (1.16–2.34)	1.60 (1.12–2.29)	1.67 (1.16–2.39)
LUH vs no HTN	1.68 (1.21–2.32)	1.45 (1.03–2.05)	1.44 (1.02–2.04)	1.47 (1.03–2.09)

*HTN* hypertension, *SCH* short duration (< 6 years) controlled hypertension, *SUH* short duration (< 6 years) uncontrolled hypertension, *LCH* long duration (< 6 years) controlled hypertension, *LUH* long duration (< 6 years) uncontrolled hypertension.

^a^The baseline model is stratified on the status of HTN.

^b^The adjusted model 1 is adjusted for confounding variables age (continuous variable), gender (male/ female), education years (continuous variable) and Occupational statues (Unemployed, Retired, Farmer, Self-employ, Employment).

^c^The adjusted model 2 has additional adjustment for confounding the variables related to habits (cigarette smoking, alcohol drinking and opium consumption), Body mass index (continuous variable) and physical activity level (continuous variable).

^d^The adjusted model 3 has additional adjustment for cholesterol (continuous variable), diabetes mellitus (yes/no), Triglycerides (continuous variable), LDL cholesterol (continuous variable), HDL cholesterol (continuous variable) and has chronic headache family (yes/no).

In the crude regression model, the odds of EPH (odds ratio (OR) 1.15, 95% CI 1.04 to 1.28) and CPH (OR 1.69, 95% CI 1.44 to 1.99) were higher among patients with HTN compared to those without HTN. HTN-EPH as well as HTN-CPH correlations continued until the confounders were adjusted in the second model. Furthermore, the corresponding adjusted ORs calculated for HTN vs. non HTN were 1.22 (95% CI 1.10 to 1.40) and 1.50 (95% CI 1.24 to 1.81), respectively for EPH and CPH (Table4).

The third adjusted model included all the variables considered in the second adjusted model plus diabetes mellitus, triglycerides, cholesterol, LDL, and HDL, which could act as potential confounders in the HTN-EPH as well as HTN-CPH associations. However, after considering new variables in the third adjusted model, the obtained results showed no more considerable changes the association of EPH (OR 1.26, 95% CI 1.12 to 1.43) and CPH (OR 1.52, 95% CI 1.25 to 1.85) with HTN.

When the results were divided by categories of HTN based on duration and control in the hypertensive patients, higher ORs were observed in the following categories; short control HTN (SCH), and Short uncontrolled HTN (SUH) with EPH; however, in CPH patients, higher ODs was found in all HTN duration and control categories.

As the use of antihypertensive drugs, such as beta-blockers, has migraine-preventing effects and may affect the prevalence of headaches, we adjusted this effect by sensitivity analysis in non-beta-blocker users. Based on the results, the odds of EPH (OR 1.28, 95% CI 1.11 to 1.48) and CPH (OR: 1.55, 95% CI 1.23–1.96) was further increased excluding recipients of beta-blocker.

## Discussion

In this cross-sectional study, we found a positive relation between EPH and HTN in participants, especially in controlled and uncontrolled HTN of short-duration groups. On the other hand, compared with EPH, CPH had a more positive relationship with HTN and all of its categories. The prevalence of HTN, EPH, and CPH was 22.5, 29.07, and 7.45%, respectively. In our cohort study, there were also general questions about EPH and CPH without mentioning their types.

The results on the prevalence of episodic and chronic headaches among our study population are similar to the results of other studies. Other studies have reported the prevalence rates of EPH and CPH to be 10–20% and almost 3%, respectively^13, 34^.

The increased prevalence of CPH could be due to the lack of perfect treatment of migraine in most migraine cases and the high prevalence of depression and stress in our country^42^. Another reason for the increase in the percentage of CPH is social and economic stress and the resulting psychological stress in our society as the global burden of disease (GBD) emphasized that primary headache disorders are an important health priority^43^.

In a study on chronic headaches that were evaluated in a headache clinic, however, the composition of the patients is different from our cohort, the results of which are slightly different from our study^44^. In headache clinics there are more migraine cases than TTH^44^. On the other hand in population-based studies, TTH is more common than migraine^45, 46^. In that study, the different percent of hypertension between migraine and TTH was not significant (7.3% versus 6.6%) but in CDH cases (16.2%) the difference is significant^46^. In our study, there was a higher percent of HTN in CPH cases than this study (31.5%versus 16.2%) and 24% of EPH in our which was more frequent than this study. Our prevalence of HTN in our headache population cases was 2 times more than this study in both groups. Our study is similar to this article in terms of the higher prevalence of hypertension in CDH than migraine and TTH as non-chronic daily headache cases^47^.

The difference between the Females/males ratio is similar to our results in CPH and EPH groups with more female prevalence in the CPH group in ours (73% versus 69%). Our results in comparing the incidence of diabetes, hypercholesterolemia, smoking, heart disease in the two groups are not different from the results of this article which were the insignificant differences^47^.

In another study, hypertension was found with a higher prevalence in TTH cases (28.71% in migraine versus 55.5%in TTH) even more than migraines cases which are in line with our results about EPH which includes more non-migraine headaches than migraine^48^. Study of Prudenzano et al. was also different from our study in that it was performed in a headache clinic but our study was on a cohort population. Another difference is more HTN prevalence in males than females in this study (54.23% versus 27.07%)^48^ which was not as our study with more HTN prevalence in females than males (p < 0.001).

There was an explanation about the higher prevalence of HTN in TTH in comparison to migraine due to antihypertensive drug use in migraine^48^, but in our study there was no significant difference between drug users and non-drug users and HTN in episodic and chronic groups. More prevalence of TTH in HTN cases in comparison to non-HTN cases may point to this idea that this type of headache might be based upon vascular mechanisms.

In a prospective cohort study of women about HTN incidence, there was higher incidence of HTN in migraines women in comparison to non-migraine women^49^. There was also more HTN incidence in Migraine without aura (21%) in comparison to Migraine with aura cases (9%). In history of migraine cases 15% incidence of HTN was seen. 18% of total population of women has migraine or history of it^49^. These findings are consistent with our results in both episodic and chronic headaches.

Furthermore, in our study, the prevalence of HTN in CPH patients was 31.98%, which is far higher than other studies such as Huang et al. study in which HTN was 27.96% prevalent in CPH patients^44^. Also, the prevalence of HTN among the cohort population (22.5%) was also less than it’s the frequency in CPH group (31.98%), while it had a prevalence rate of 24.58% in the EPH group. These findings are consistent with other studies^45, 50^.

An epidemiological studies carried out in Nord-Trondelag Health Survey also revealed a strong negative relation between HTN and Migraine, while the Northern Manhattan study described a strong correlation between HTN and Migraine in controlled and uncontrolled HTN of long duration groups as well as in short duration uncontrolled, a weaker correlation^26, 51^. The prevalence of Migraine in a study performed in the Northern Manhattan was 20.40% which is less than what we found in the present work about EPH^26^. Unlike the current research, the Northern Manhattan study found a strong HTN-Migraine correlation in controlled and uncontrolled HTN of short duration groups of cases.

Race could be an effective factor, which has led to these differences. For example, Hispanics have shown stronger long duration HTN correlation with Migraine than Caucasian. Environmental factors, life styles, and occupational factors have also proved to be able to influence hypertension and Migraine^26^. Moreover, in HTN categories, prolonged use of some antihypertensive drugs, such as angiotensin converting enzyme inhibitors and angiotensin receptor blockers may have been the reason for reducing Migraine in long duration HTN cases which was not confirmed in our study.

In line with our findings, an Italian cohort study presented that young females had a higher chance to catch Migraines^51^. This Italian study also verified that Migraine is associated with HTN and tension-type headaches. However, they reported a strong association between tension-type headaches and myocardial ischemia. Furthermore, some studies have described an association between chronic kidney disease (CKD) and Migraine so that older patients with Migraine showed a higher incidence of CKD. Furthermore, using non-steroid anti-inflammatory drugs (NSAIDs) and prophylaxis drugs in Migraines have shown a high risk of HTN^51^. In addition, our findings of HTN categories matched with CPH cases but not as with Migraine cases in the Gardner et al. study^26^. Although there was many articles in favor of linking HTN to migraines and other primary headaches, the link is still unclear^21^.

One of the main strengths of our study is its population-based nature with a large sample size, extensive data collection for the exposure of interest (hypertension) and potential confounders. Another advantage was the new comparison between chronic and episodic primary headaches in relation to hypertension.

### Limitations

The findings of the present research work might have been influenced by different kinds of bias, such as cross-sectional design, imprecise response about previous headache or diagnosis of Primary episodic headache, and wrong recall of subject’s Primary episodic headache information. One limitation of our study is that some of the antihypertensive medications have migraine preventive effects and may affect the prevalence of headaches in our study. In this regard, we performed sensitivity analysis by for exclusion of beta blocker-users, which showed a further increased odds ratio of EPH and CPH associated with HTN.

Another limitation of our cohort study is the lack of highly detailed questions to obtain sufficient information to be able to sub-classify the primary headaches in episodic and chronic types. The reason has been to keep the length of the interview sessions in a standard tolerable time-range. Future studies are warranted to assess the sub-classes of primary headaches in relation to hypertension. We suggest adding extra questions to the questionnaires for the follow-up phase of the PERSIAN cohort to address this limitation.

We offer adding these information about types of primary headaches in follow-up phase of Persian cohort participants and another study about relation of sub classification types of primary headaches with hypertension in another prospective study.

## Conclusions

Our study showed high prevalence of HTN in CPH and EPH cases. Also, unemployed as well as high-BMI patients, who were not cigar, opium, or alcohol users, demonstrated higher risk of EPH or CPH. All HTN categories showed higher prevalence of CPH in terms of duration and control than EPH, which only displayed strong correlation with controlled HTN which has related more with controlled HTN categories.

Finally, further clarification of the EPH-HTN relationship is necessary through performing prolonged cohort studies on younger patients divided in two groups of HTN and non-HTN to compare the prevalence of EPH and CPH between them.

## Data Availability

The datasets used during the current study are available on the Persian Adult Cohort Study Center, Rafsanjan University of Medical Sciences, Iran. The data is not available publicly. However, upon a reasonable request, the data can be obtained from the authors.

## Abbreviations

RCS: The Rafsanjan cohort study
PERSIAN: Prospective epidemiological research studies in Iran
EPH: Episodic primary headaches
CPH: Chronic primary headache
HTN: Hypertension
CVDs: Cardiovascular diseases
CDH: Chronic daily headache
CM: Chronic Migraine
YLDs: Years lived with disability
ICHD: International Classification of Headache Disorders
SBP: Systolic blood pressure
DBP: Diastolic blood pressure
AH: Arterial hypertension
TTH: Tension-type headache
CTTH: Chronic tension type headache
MOH: Medication overuse headache
LDL: Low-density lipoprotein
HDL: High-density lipoprotein
BMI: Body mass index
PA: Physical activity
MET: Metabolic equivalent of task
SD: Standard deviation
OR: Odds ratio
CKD: Chronic kidney disease
NSAIDs: Non-steroid anti-inflammatory drugs
SCH: Short duration (< 6 years) controlled HTN
SUH: Short duration (< 6 years) uncontrolled HTN
LCH: Long duration (≥ 6 years) controlled HTN
LUH: Long duration (≥ 6 years) uncontrolled HTN
